# Identification of a five B cell-associated gene prognostic and predictive signature for advanced glioma patients harboring immunosuppressive subtype preference

**DOI:** 10.18632/oncotarget.12605

**Published:** 2016-10-12

**Authors:** Chuanbao Zhang, Jiye Li, Haoyuan Wang, Sonya Wei Song

**Affiliations:** ^1^ Beijing Neurosurgical Institute, Capital Medical University, TiantanXili, Dongcheng District, Beijing 100050, China; ^2^ Beijing Institute for Brain Disorders, Youanmen, Beijing, 100069, China; ^3^ Center for Brain Disorders Research, Capital Medical University, Youanmen, Beijing, 100069, China; ^4^ Department of Neurosurgery, Zhujiang Hospital, Southern Medical University, Guangzhou, 510282, China

**Keywords:** immune system, tumor-infiltrating B cells, prognosis, biomarker, glioma

## Abstract

High grade gliomas contribute to most brain tumor mortality. A few studies reported that the immune system affected glioma development, and immune biomarkers helped understand the disease and formulate effective immunotherapy for patients. Currently, no B lymphocyte-based prognostic signature was reported in gliomas. By applying 78 B cell lineage-specific genes, we conducted a whole-genome gene expression analysis in 782 high grade gliomas derived from three independent datasets by Cox regression analysis and risk score method for signature identification, and then used Gene Ontology, Gene Set Enrichment Analysis, and other statistical methods for functional annotations of the signature-defined differences. We developed a five B cell-associated gene signature for prognosis of high grade glioma patients, which is independent of clinicopathological and genetic features. The signature identified high risk patients suitable for chemoradiotherapy, whereas low risk patients should rule out chemotherapy with radiotherapy only. We found that tumors of TCGA Mesenchymal subtype and wild type *IDH1* were preferentially stratified to the high risk group, which bore strong immunosuppressive microenvironment, while tumors of TCGA Proneural subtype and mutated *IDH1* were significantly accumulated to the low risk group, which exhibited less immunosuppressive state. The five B cell-associated gene signature predicts poor survival of high risk patients bearing strong immunosuppression and helps select optimal therapeutic regimens for glioma patients.

## INTRODUCTION

Malignant glioma is the most common primary brain tumor and has inevitable local recurrence, thus contributing to a considerable brain tumor-related mortality in adults and children. High grade gliomas (HGGs) (World Health Organization WHO grade III and grade IV) consist of glioblastoma (GBM) (WHO grade IV) and anaplastic gliomas (WHO grade III) including anaplastic astrocytoma (AA), anaplastic oligodendroglioma (AO), and anaplastic oligoastrocytoma (AOA) [1, 2]. The standard therapy for the disease includes surgery, radiotherapy (RT), chemotherapy (CT), and chemoradiotherapy (CRT) [3]. Despite the improvements in these therapies, the median survival of the patients with GBM and anaplastic gliomas is only 15 months and three years respectively [4]. Moreover, highly variable prognosis exists in HGGs such as 3–5% of GBM patients surviving longer than three years, thus hindering precise patient stratification and treatment [5]. This is mainly because of a heterogeneous population of the HGG tumors and their variable microenvironments, which affect tumor progression and patient survival [6].

Tumor-infiltrating immune cells have been recognized as an essential factor for clinical outcomes of cancer patients [7]. CD8+ cytotoxic T lymphocyte infiltration has been found to be associated with favorable outcomes in many cancer types such as non-small cell lung cancer (NSCLC), glioma, esophageal and rectal cancer [8–11]. However, the tumor-infiltrating immune cells are frequently modulated by the local cellular and soluble components of the tumor microenvironment, resulting in the generation of inhibitory immune cells. Immunosuppressive T regulatory cells (Treg), myeloid-derived suppressor cells (MDSC), and tumor-associated M2 macrophages are commonly present in the tumor mass, which create the immunosuppressive microenvironment and support tumor growth and progression [12]. Therefore, the intra- and peritumoral presences of immune infiltrates significantly impact patient survival [13].

B lymphocyte is recently recognized to participate in regulating immune response to murine and human tumors. A subset of B cells, regulatory B cells (Breg), plays an immunosuppressive role in carcinogenesis and becomes a therapeutic target in solid tumors [14, 15]. Some findings also indicate that B cell-mediated immune response or associated tumoral tertiary lymphoid structure is favorable for patient survival in NSCLC and hepatocellular carcinoma [16, 17]. At present, few studies explore the role of tumor-infiltrating B cells in malignant glioma and their impact in clinical outcomes of the patients remains unknown.

In the study, we applied whole-genome mRNA expression profiles of gliomas and a set of 78 B cell lineage-specific genes to identify a B cell-specific signature for clinical outcomes of HGG patients. We found a five B cell lineage-specific gene signature (four risky genes of BACE2, FCGR2B, ISG20, and SWAP70 and one protective gene of QRSL1), which successfully stratified HGG patients into a high risk group with poorer survival and a low risk group with better outcomes and helped select optimal adjuvant therapies for HGG patients. The findings support the possibility that treatment strategies targeting HGG-infiltrating immunomodulatory cells are therapeutically beneficial.

## RESULTS

### Identification of a five B cell-associated gene signature for prognosis in high grade gliomas independent of clinicopathological and genetic features

**Figure 1:**
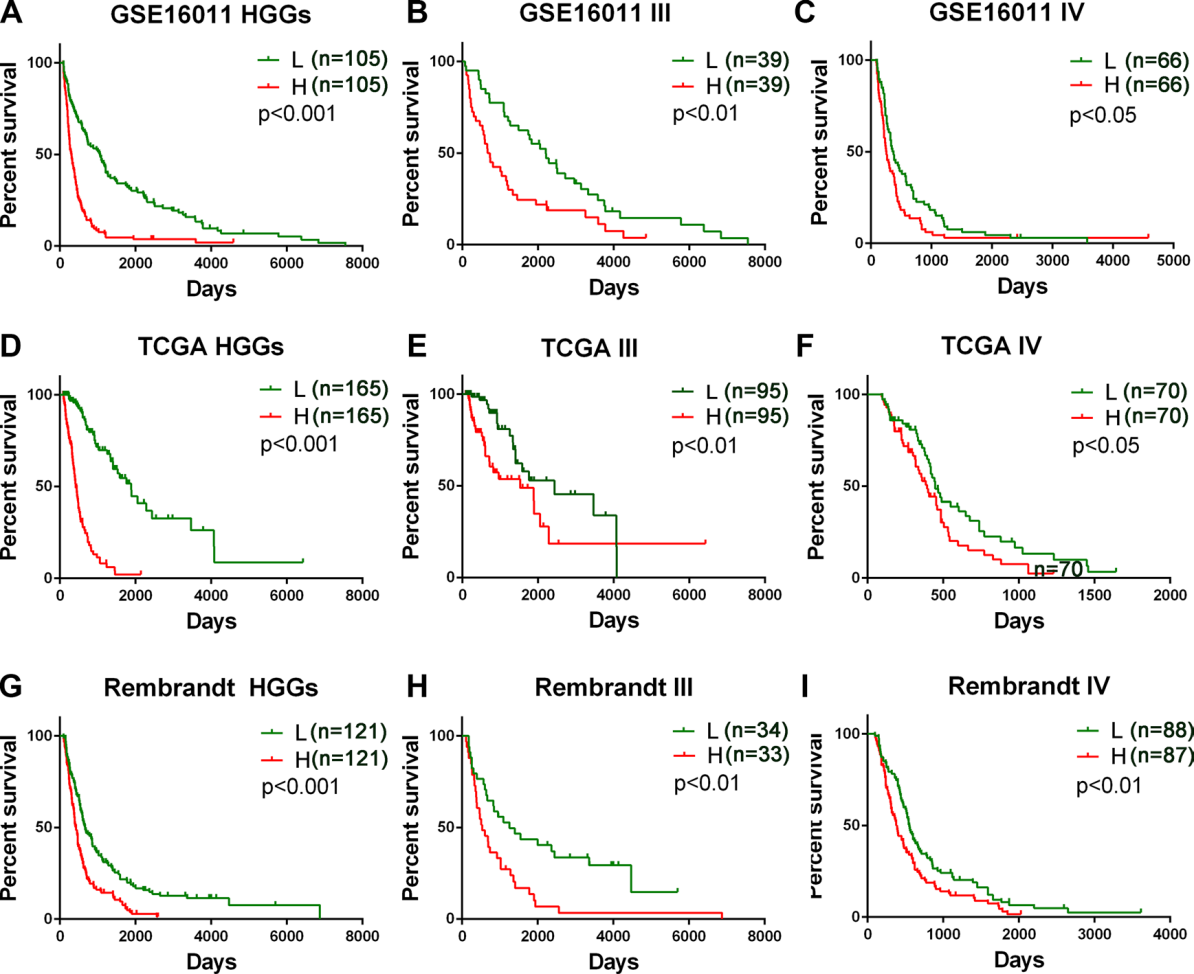
Prognostic value of the five B cell-associated gene signature for glioma patients in training and validation datasets Patients in the low risk group showed a better survival than those in the high risk group according to the signature risk score in GSE16011 dataset (**A**–**C**), TCGA dataset **D**–**F**), and Rembrandt dataset (**G**–**I**). L, low risk group; H, high risk group; III, WHO grade III; IV, WHO grade IV; HGGs, high grade gliomas.

**Figure 2:**
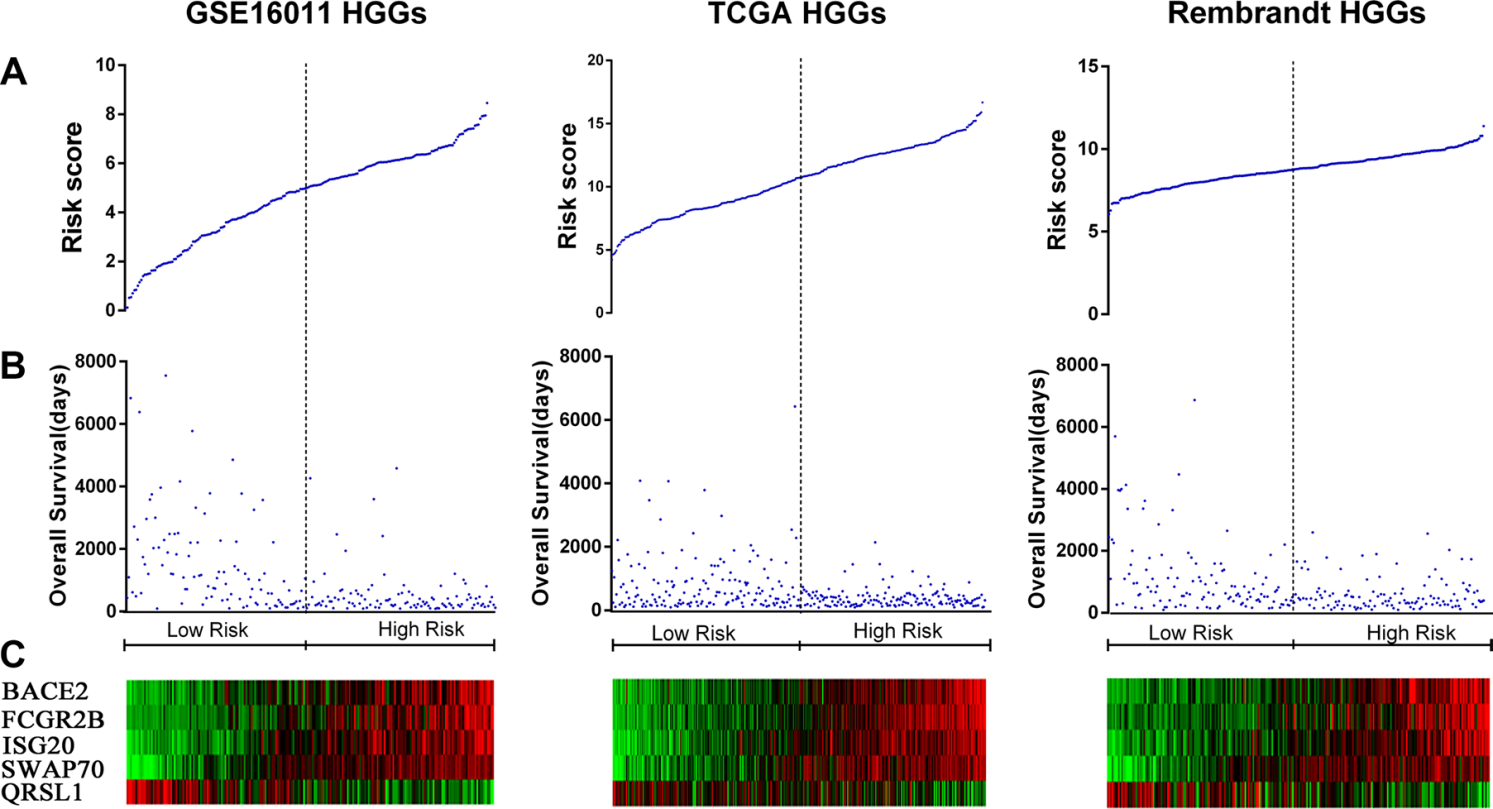
Distribution of risk scores of HGGs and OS of their patients based on the five signature genes in the three datasets (**A**) Risk score distribution among HGGs. (**B**) Patient overall survival among HGGs. (**C**) Expression of five signature genes among HGGs.

To identify a B cell-associated gene prognostic signature in gliomas, we first assessed expression patterns of 78 B cell lineage-specific genes among glioma grades by Student's t test and then calculated prognostic values of differentially expressed genes by univariate Cox regression analysis using gene expression profiles of GSE16011 dataset as a training set. Five differentially expressed B cell-specific genes were identified to be significantly associated with overall survival (OS) of HGG patients (*P <* 0.001, Table 1). The five significant genes were classified into two types of genes: risky and protective genes. Risky and protective genes were defined as ones that had hazard ratios for death greater and less than 1 respectively. Using this definition, we found four risky genes (BACE2, FCGR2B, ISG20, and SWAP70) and one protective gene (QRSL1). To test the predictive power of the five genes as a signature, we developed a risk score formula by using a linear combination of the expression levels of the five genes weighted with their regression coefficients as described in Methods. The risk score for each patient was then calculated in grade II, III, IV, and HGG gliomas. Using the median risk score as the cutoff value, the patients were successfully divided into a high risk group and a low risk group in each grade (Figure 1A–1C, Supplementary Figure S1A). The patients with the high risk score had a shorter median OS than ones with the low risk score especially in HGGs (*p <* 0.001) (Figure 2A and 2B). Hierarchical clustering of the five gene expression in HGG tumors showed that tumors of high risk patients expressed high levels of the risky genes and a low level of the protective gene (Figure 2C) (*p <* 0.001). Also the risk gene expression increased and the protective gene expression decreased with increased malignancy of gliomas consistent in the three datasets (Supplementary Figure S2).

**Table 1:** Five B cell-associated genes were significantly associated with overall survival of HGG patients in GSE16011 dataset

Symbol	Hazard radio	95% Confidence interval	Parametric *p* value
BACE2	1.436	(1.281, 1.609)	4.90E-10
FCGR2B	1.246	(1.146, 1.355)	2.58E-07
ISG20	1.633	(1.420, 1.879)	6.29E-12
QRSL1	0.313	(0.219, 0.448)	1.75E-10
SWAP70	1.694	(1.452, 1.975)	1.83E-11

We then determined the dependence of the signature of clinicopathological and molecular parameters in HGG patients from GSE16011 dataset by univariate and multivariate Cox regression analyses. All the parameters (Table 2) were selected based on our clinical experience that were related to prognosis. We found that the signature (risk score), age, preoperative KPS score, *IDH1* status, and histology were statistically associated with OS (*p <* 0.01) (Table 3). Multivariate Cox analysis indicated that the signature was an independent prognostic factor (*p* = 0.018) after adjusted for age, preoperative KPS score, and histology (Table 3).

**Table 2:** Clinicopathological and molecular parameters of HGG patients in GSE16011 dataset (*n* = 210)

Variable		Total	Low risk score (*n* = 105)	High risk score (*n* = 105)	*p* value
Age at diagnosis	< 45	74	50	24	< 0.05
	≥ 45	136	55	81	
Gender	Male	139	64	75	> 0.05
	Female	71	41	30	
Preoperative KPS score	< 80	57	28	29	> 0.05
	> 80	144	72	72	
	NA	9	5	4	
Histology	AA	13	11	2	< 0.05
	AO	42	33	9	
	AOA	23	20	3	
	GBM	132	41	91	
*IDH1* mutation	Mut	59	42	17	< 0.05
	WT	108	45	63	
	NA	43	23	21	
Extent of surgery	Total	67	32	35	> 0.05
	Subtotal	114	64	50	
	NA	29	12	17	
Radiotherapy	Yes	175	86	89	Not available
	No	0	0	0	
	NA	35	19	16	
Chemotherapy	Yes	26	13	13	> 0.05
	No	150	75	75	
	NA	34	17	17	

**Table 3:** Factors associated with overall survival of HGG patients by Cox regression analysis in GSE16011 dataset

Variable	Univariate Cox Regression			Multivariate Cox Regression		
	HR	95%CI	*p* value	HR	95%CI	*p* value
***Overall Survival***						
Gender (Male vs. Female)	1.010	0.752–1.356	> 0.05			
Age at diagnosis(< 45 vs. > 45)	2.661	1.945–3.639	< 0.001	2.417	1.666–3.507	< 0.001
Preoperative KPS score(> 80 vs.< 80)	0.577	0.419–0.792	< 0.01	0.532	0.456–1.044	< 0.01
Risk score (Low vs. High)	2.749	2.038–3.708	< 0.001	1.603	1.082–2.372	0.018
*IDH1* status (Mut vs. WT)	0.449	0.319–0.633	< 0.001	0.672	0.451–1.002	0.051
Histology*	1.622	0.916–1.146	< 0.001	1.435	1.217–1.693	< 0.01
Chemotherapy (Yes vs.No)	0.640	0.408–1.005	0.052			
Extent of surgery(Total vs. Subtotal)	0.905	0.662–1.239	> 0.05			

* Histology was defined as 1, AO, 2, AOA, 3, AA; 4.GBM.

Currently, there are few reports related to the five genes in the field of Neuro-oncology. *BACE2*, a member of beta-site APP-cleaving enzyme family of genes, encodes an integral membrane glycoprotein that functions as an aspartic protease related to Alzheimer's disease [18]. *FCGR2B* (*CD32B*)-encoded protein is a member of Fc receptor common γ chain (FcRγ) family containing an immune tyrosine-based inhibitory motif (ITIM), which is a low affinity receptor for the Fc region of IgGs and down-regulates the antibody production by B cells. It is a promising therapeutic target for malignancies [19, 20]. *ISG20* encodes an exoribonuclease that acts on a single-stranded RNA, exhibits an antiviral activity against RNA viruses in an exonuclease-dependent manner, and probably plays accessory roles in the maturation of snRNAs and rRNAs [21]. *SWAP70*-encoded protein specifically binds to phosphatidylinositol-3, 4, 5-triphosphate, transduces signals from tyrosine kinase receptors to RAC-protein kinase B (RAC/PKB), and regulates signaling of membrane ruffling. Studies have shown that SWAP70 is involved in signaling B cell activation and may have a potential oncogenic function in cancer [22–24]. *QRSL1*-encoded protein allows the formation of correctly charged Glu-tRNA through the transamidation of misacylated Glu-tRNA (Gln) in the mitochondria in the presence of glutamine and ATP through an activated gamma-phosphor-Glu-tRNA [25].

### Validation of the B cell-associated gene prognostic signature in two independent HGG platforms

To validate the prognostic power of the signature, we used two independent glioma mRNA expression profiling datasets from Rembrandt and TCGA RNAseq databases. In 242 HGGs of 311 glioma cases from REMBRANDT and 330 HGGs of 502 glioma cases from TCGA, we applied the same risk score formula developed in the training set to calculate the risk score for each patient and accordingly divided the patients into the low and high risk groups in line with the risk score using the same cutoff. Similarly as in the training set, the patients were successfully stratified into the high and low risk groups with the high risk patients having a shorter OS (*p <* 0.001) (Figure 1D–1I, Supplementary Figure S1B and S1C, Figure 2A and 2B) and expressing the high levels of the risky genes and the low level of the protective gene (Figure 2C) (*p <* 0.001).

### The B cell-associated gene signature assisted predicting the efficacy of radiotherapy and chemoradiotherapy for low and high risk HGG patients

**Figure 3:**
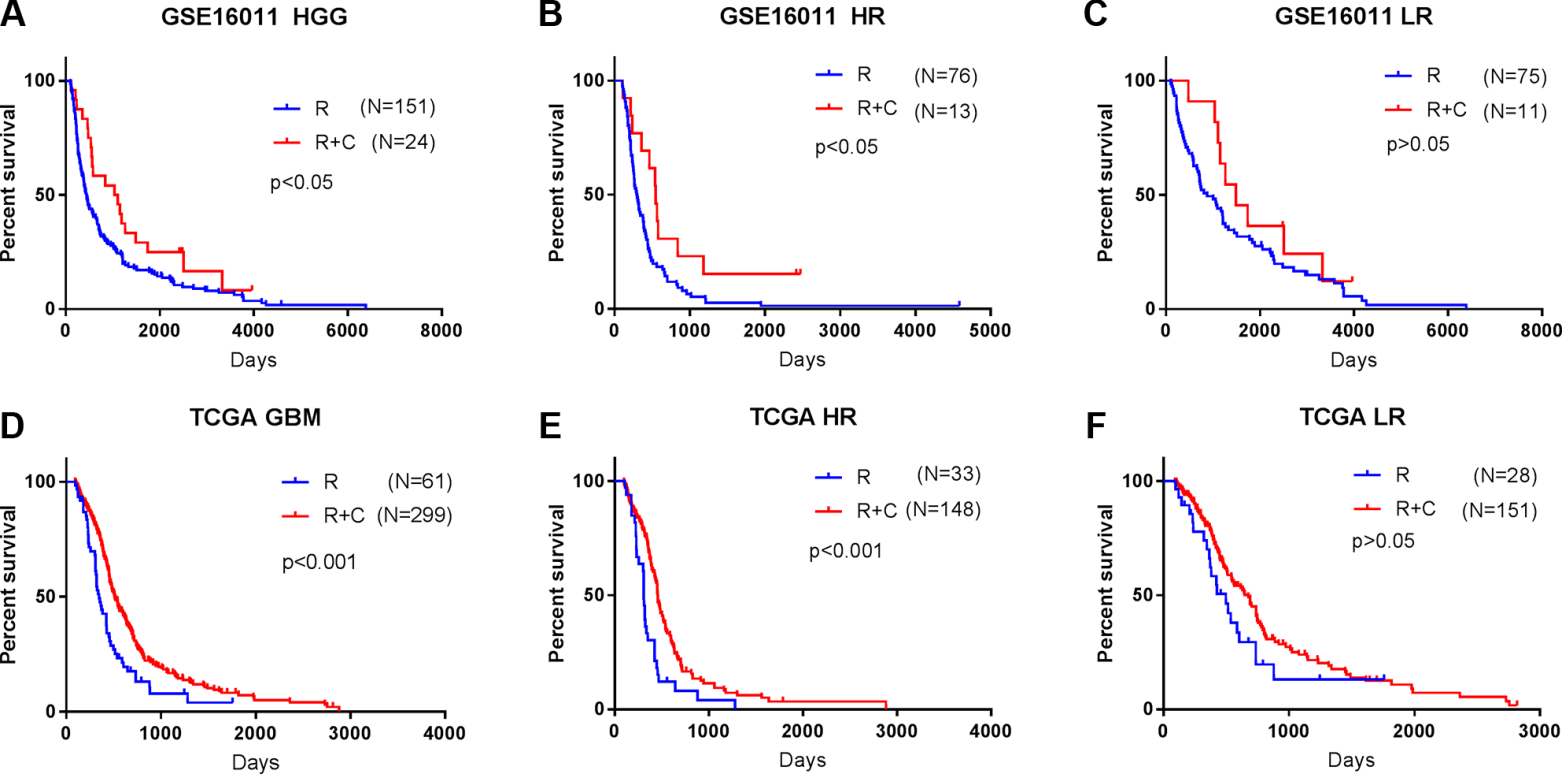
The signature assisted predicting the efficacy of radiotherapy with or without chemotherapy in HGG patients (**A**, **D**) HGG patients in GSE 16011 and GBM patients in TCGA treated with CRT showed a better prognosis than those with RT alone. (**B**, **E**) Benefit of CT was observed in the high risk group with significantly improved OS (*p <* 0.05). (**C**, **F**) The addition of CT to RT did not improve OS of patients in the low risk group (*p* > 0.05). R, radiotherapy; R + C, radiotherapy + chemotherapy; LR, low risk group; HR, high risk group.

To determine whether the signature assists predicting the efficacy of the postoperative RT and CT in HGG patients, we extracted the therapeutic information available for 175 HGG patients in GSE16011 dataset. According to their risk scores calculated using the signature, 89 patients (76 patients underwent RT and 13 patients underwent CRT) were stratified to the high risk group and the other 86 patients (75 patients underwent RT and 11 patients underwent CRT) to the low risk group. We then compared the survival advantage between RT and CRT by Kaplan-Meier plotting in each group. We found that OS did not differ significantly between RT and CRT in the low risk group (*p >* 0.05), that is, the addition of CT to RT did not improve OS of HGG patients with the low risk scores, but the benefit of CRT was observed in the high risk group with significantly improved OS (*p <* 0.05) (Figure 3A–3C).

We then used 360 GBM patients treated with standard RT with or without temozolomide (TMZ) chemotherapy in TCGA dataset to confirm the therapeutic predictive value of the signature. Similarly, CRT was only beneficial for the high risk GBM patients (*p <* 0.001) (33 RT/148 CRT) but not for the low risk GBM patients (*p >* 0.05) (28 RT/151 CRT) (Figure 3D–3F). The findings suggest that the low risk patients should avoid unnecessary chemotherapy.

### The signature-stratified low and high risk HGGs exhibited distinct molecular features

**Figure 4:**
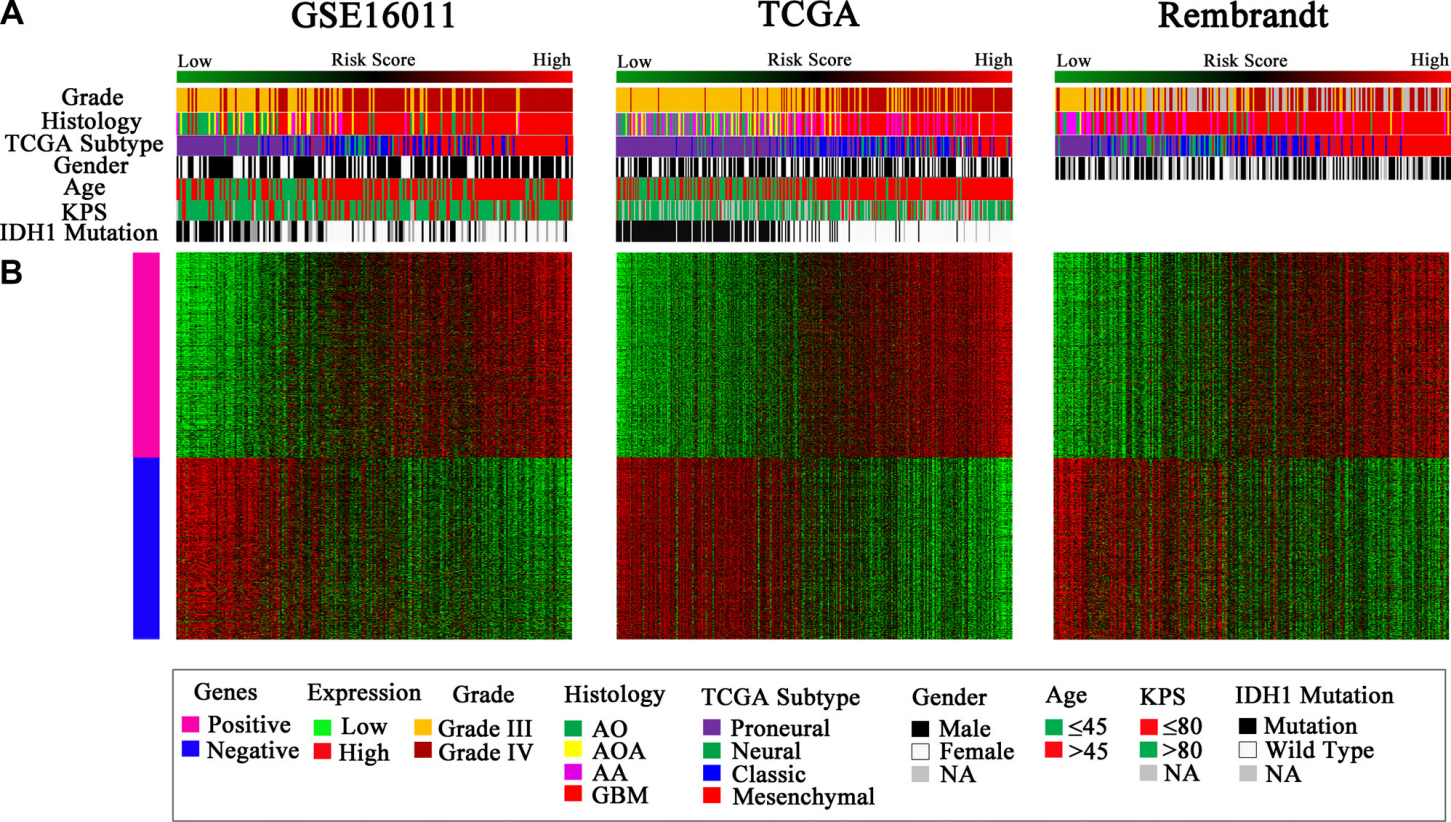
Distribution of molecular and clinicopathological features for HGGs and their patients aligned with the risk score in the three datasets (**A**) Tumors with high risk scores contained almost all TCGA Mesenchymal subtype and wild-type *IDH1*, and tumors with low risk scores included most of TCGA Proneural subtype and mutated *IDH1*. Grade III and IV tumors were distributed in both the low and high risk groups. (**B**) The differentially expressed genes were shown from the low to high risk score tumors. Pink represents the high expression of genes in the high risk group; blue represents the low expression of the genes in the high risk group.

**Figure 5:**
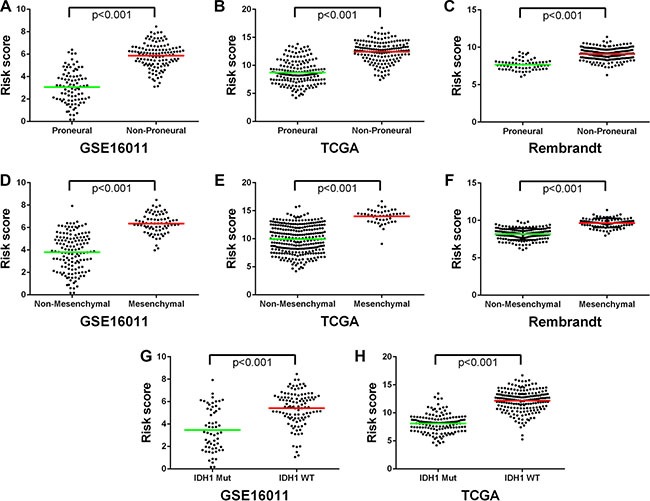
The low and high risk score HGGs exhibited distinct TCGA molecular subtypes and *IDH1* mutation status (**A**–**C**) The Proneural subtype was preferentially stratified into the low risk group. (**D**–**F**) The Mesenchymal subtype was mainly stratified to the high risk group. (**G**, **H**) The *IDH1* mutation was dominant in the low risk group while the wild-type *IDH1* preferentially present in the high risk group. Each spot represents the risk score of the individual HGG. Line in the middle was the mean value of the risk score.

Considering the potential of the signature in predicting clinical therapies, we next assessed the association of the low and high risk groups with some known molecular features of gliomas and related clinical characteristics. We found that tumors with high risk scores included almost all TCGA Mesenchymal subtype tumors and wild-type *IDH1*, whereas tumors with low risk scores contained most of TCGA Proneural subtype tumors and *IDH1* mutation consistently in the three datasets of GSE16011, TCGA, and REMBRANDT (*P <* 0.001) (Figure 4A, Figure 5A–5H). However, the signature stratified TCGA Classical and Neural subtype tumors, as well as grade III and IV tumors described earlier (Figure 1), into the low and high risk groups (Figure 4A), patients of which showed differential survival advantages (Supplementary Figure S3) in TCGA dataset.

### The high risk HGGs exhibited enhanced expression of immunosuppressive factors and regulatory immune cells

**Figure 6:**
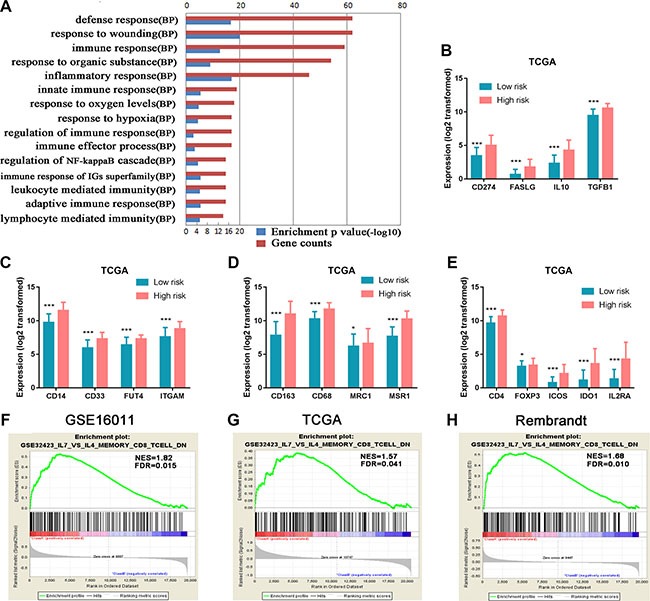
Functional annotation of the high risk versus the low risk group (**A**) GO analysis revealed that 416 genes with increased expression in the high risk group were mainly related to immune response. Red column height: gene counts; blue column height: enrichment *p value*. BP, biological process. (**B**) Genes encoding immunosuppressive factors *TGF-β, IL10, CD274* (protein name PDL1), and *FASLG* (FASL) were highly expressed in the high risk group. (**C**, **D**) MDSC marker genes (*ITGAM* (CD11b), *CD14, FUT4* (CD15), and *CD33*) and M2 microglia/macrophage marker genes (*CD68, CD163, MSR1* (CD204), and *MRC1* (CD206)) were significantly up-regulated in the high risk group. (**E**) Tregs marker genes (*CD4*, *IL2RA*(CD25), *ICOS, IDO1*, and *FoxP3*) showed increased expression in the high risk group. (**F**–**H**) The enrichment plots of the gene expression related to inhibiting activation and differentiation of CD8^+^ T cells to cytotoxic CD8^+^ T cells were separated between the low and high risk score groups. Class A, the high risk group; Class B, the low risk group; NES, Normalized Enrichment Score; FDR, False Discovery Rate.*P < 0.05; **P < 0.01; ***P < 0.001.

The notable differences in the two risk groups of HGGs led us to further conduct a whole-genome gene expression analysis by SAM method using the same three datasets to obtain a broader biological insight between the two groups. After 1000 times of permutation test, those genes with FDR < 0.05 were considered to be differentially expressed between the two groups, which exhibited a good separation from the low to high risk score shown by one-dimensional hierarchical clustering analysis (Figure 4B). By screening top 1000 increased expression genes and 1000 decreased expression genes in the three datasets, the overlapped genes (416 genes with increased expression and 368 genes with decreased expression) in the high risk group (Supplementary Table S1B and S1C) were chosen for further analysis. The positively correlated genes (pink marked genes in Figure 4B) were used for GO analysis. The top 15 GO terms indicated that these genes were mainly related to immune response such as defense response, inflammatory response, positive regulation of I-kappaB kinase/NF-kappaB cascade, leukocyte-mediated immunity, and response to hypoxia and oxygen levels (Figure 6A).

HGG patients generally have poor immune response. To further decipher the association of the immunological state with HGGs, we specifically analyzed immunosuppressive gene expression between the low and high risk groups in TCGA dataset. Immunosuppressive factor genes (*IL10*, *TGFβ1*, *CD274* (protein name PDL1) and *FASLG* (FASL)) were significantly increased in the high risk group compared with the low risk group (Figure 6B). Marker genes of immunosuppressive cells such as myeloid-derived suppressor cells (MDSC) (*ITGAM* (CD11b), *CD14*, *FUT4* (CD15), *CD33*) [26] and M2 microglia/macrophages (*CD68*, *CD163*, *MSR1* (CD204) and *MRC1* (CD206)) [27, 28] were significantly up-regulated in the high risk group compared with the low risk group (Figure 6C and 6D). Similarly, the expressions of Treg marker genes (*CD4, ICOS, IDO1, FoxP3, IL2RA* (CD25)) [29] were elevated in the high risk group (Figure 6E). In addition, we also observed the similar expression patterns of the immunosuppressive factors and cell markers in the other two datasets of GSE16011 and Rembrandt (Supplementary Figure S4). GSEA analyses of the three datasets further indicated that the high risk group had increased expression of genes (Supplementary Table S1D) that inhibit the activation of memory CD8+ T cells and their differentiation to cytotoxic CD8+ effector T cells (Figure 6G–6I).

Taken together, the findings demonstrated that strong immunosuppressive tumor microenvironment contributed to the worse malignancy of the tumors and eventually poorer survival of the patients stratified in the high risk group.

## DISCUSSION

A growing body of evidences has supported the interaction between the immune system and glioma pathogenesis [30, 31]. A few prognostic immune signatures related to T cell, NK cell, and microglia/macrophage have been reported for gliomas [32, 33], but no B cell-associated signature was studied in the disease. Here, by using a risk score method we identified and validated a signature composed of five B cell associated genes based on the three independent genome-wide gene expression datasets. The signature successfully divides glioma patients especially high grade glioma patients into the low risk group with favorable OS and the high risk group with poor survival, consistently the latter exhibiting significantly strong immunosuppressive microenvironment in their tumors. The signature also shows that the addition of chemotherapy to radiotherapy improves OS of the high risk patients, but not that of the low risk ones after surgery. These findings aid in better understanding of the interplay between immune response and glioma progression.

The immune responsive microenvironment of tumors contributes to an antitumor activity while the immune suppressive state promotes tumor development [34]. In our study, we have found that the major differences of the low and high risk groups of the HGG patients are related to immune response such as inflammatory response and leukocyte-mediated immunity. Because the microenvironment of GBM is highly immunosuppressive [35], we have then analyzed the gene expression patterns of the two risk groups and found that the high risk group exhibits significantly increased expression of classical immunosuppressive factors such as IL10, TGF-β, PDL1, and FASL and many immunosuppressive cell markers related to immunosuppressive cells such as tumor-associated macrophage M2, Tregs, and MDSC. The presence of immunosuppressive factors such as IL-10 and TGF-β and inhibitory molecules on the GBM cell surface were reported to inhibit the antitumor activities of T cells, B cells, NK cells, and monocytes [35]. FASL was expressed in human malignant glioma cells and induced apoptosis of T lymphocytes [36]. Astrocytoma cell lines expressed PDL1, which inhibited T cell functions such as proliferation and cytotoxicity and promoted apoptosis [37]. In addition, the presence of immunosuppressive infiltrates such as FoxP3+ Treg cells, M2 macrophages, and MDSC were documented in gliomas and associated with poor survival in gliomas [38, 39]. These studies support our findings that tumors of the five B cell-associated gene signature-stratified high risk group are more immune suppressive compared with those of the low risk group, thus creating a more favorable microenvironment for glioma progression.

Accurate classification of tumors is important for appropriate treatment selection. Nevertheless, no single genetic alteration could elucidate the complicated pathogenesis of HGGs, leading to the difficulty in choosing appropriate therapeutic strategies for HGG patients. In the study, we found that the addition of CT to RT did not improve OS of patients in the low risk group compared to the benefit of CRT in the high risk group with significantly improved OS. Accumulated evidence indicates that RT and CT potentiate an antitumor activity in esophageal and rectal cancer via activating immune response through increased tumor antigen exposure by CT-induced immunogenic tumor cell death and released proinflammatory cytokines to activate T effector cell response [40, 41]. In addition, preclinical studies have demonstrated that the density of CD4+ and CD8+TILs is positively associated with good response after RT, CT and CRT [42]. Furthermore, basic studies of breast and prostate cancer suggest that RT or CT may be more sufficient in immunocompetent mice than immunosuppressive ones [43, 44]. We have found that the addition of CT to RT can not significantly improve OS in the low risk group. This is probably because of good immune responsive state of those patients and thus having good response to RT, in other words, RT only is sufficient to generate the same therapeutic effect as CRT, meanwhile avoiding the toxicity of adjuvant CT. However, we have found that the addition of CT generates the better outcomes for the high risk patients with strong immunosuppression. This is most likely because CT-induced myelosuppression and lymphopenia effectively deplete immunosuppressive cells such as Treg cells and thus eliminate the immune tolerance to autologous tumor antigens [45–47] in the group of patients. Such lymphodepletion induces reactive homeostatic proliferation and generates more active immune response to tumor antigens from CRT-caused tumor cell death [48, 49], leading to significantly improved OS of this group of patients. Therefore, the signature can identify glioma patients with a poor survival who may take advantage of adjuvant CT and patients with a favorable survival who should avoid the CT treatment.

More than 70% of gliomas carry the mutation of *IDH1* (R132H), which occurs in the critical arginine residue (Arg, R) in the catalytic pocket to histidine (His, H) [50]. Recent studies have shown that *IDH1* (R132H) represents an immunogenic tumor antigen recognized by CD4+IFN-γ-producing T cells in patients [51]. In our study, the *IDH1* (R132H) mutation was preferentially found in the low risk group with the more immune responsive state. Basic studies showed that an *IDH1* (R132H) vaccine induced a specific antitumor immune response against *IDH1* (R132H)-mutated tumors and the antitumor effect was associated with CD19+B cells through their antigen presentation capacity in an MHC-humanized mice model [52]. Coincidently, we have found that the high expression of *FCRG2B* (*CD32B*), the inhibitor of antigen presentation and antibody production by B cells, occurs in the high risk group, which was opposite with the preferential occurrence of *IDH1* mutation in the low risk group. Our previous study showed that the plasma of patients of low grade tumors contained a higher level of IgG autoantibodies against tumor-associated antigen IGFBP2 than that of high grade tumor patients [53].

The molecular heterogeneity of gliomas especially GBM has been widely recognized. Four molecular subtypes (Proneural, Neural, Classical, and Mesenchymal) were identified and adopted by TCGA [54]. The correlation between these subtypes with the immune system has attracted increased attention because of increasingly recognized roles of the immune system in etiology and developing new immunotherapy in gliomas. Previous studies showed the existence of different immunological states between Proneural and Mesenchymal subtypes [55], and an immune prognostic signature was reported for GBM patients especially for ones with the Proneural subtype [56]. We have found that the patients with the lower risk score are preferentially associated with the Proneural subtype and exhibit a significantly better prognosis, and the patients with the higher risk score show the Mesenchymal preference and poor survival, which are consistent with the previous report showing that Mesenchymal gliomas with immune suppressive nature were more aggressive and led to poor patient survival [57].

The role of tumor-infiltrating B cells (TIL-Bs) in gliomas remains poorly understood. Some studies reported B cell infiltration in gliomas [58, 59]. Engler et al. found that microglia/macrophage-related genes were significantly enriched in the Mesenchymal subtype compared to the non-Mesenchymal ones, but the B cell gene signature was not enriched to the significance between the two groups [59], which is likely due to impaired normalized enrichment score of the B cell gene set signature when the whole B cell-specific gene set including ones not directly associated with the disease is incorporated in the enrichment analysis. In other cancer types such as breast cancer and NSCLC, TIL-Bs can act as antigen presenting cells (APCs) for a variety of tumor antigens and interplay with CD4+ and CD8+ T cells for increased survival [60, 61]. In a GBM mouse model, B cells act as APCs for T cell-mediated antitumor immunity and tumor regression [62]. However, the role of B cells in tumor immunity has remained controversial. Other reports demonstrated that TIL-Bs suppressed immune response in some tumor types such as lymphoma, colon cancer, melanoma, and skin carcinoma [63, 64]. In the study, we have found that the risky gene *FCGR2B* is highly expressed in tumors of the high risk group, which was reported to play the immunosuppressive role, although the functions of other four signature genes in the immune system remain to be explored. Therefore, based on our findings, *FCGR2B* could be a target for immune checkpoint inhibition to improve antitumor response of immunotherapy for glioma patients.

In summary, our study has provided a clear view that the immune system such as the B lymphocyte interplays with gliomas and thus influences the prognosis of HGG patients. To our knowledge, this is the first report to identify a signature composed of the B cell-associated genes, which reveals different immunological states of glioma tumors of the low and high risk patients. The signature can help stratify HGG patients for optimal treatment strategies.

## MATERIALS AND METHODS

### Databases and B cell lineage-specific genes used in the study

Whole-genome mRNA expression microarray data and clinical information (including age at diagnosis, gender, preoperative Karnofsky Performance score (KPS), histology, postoperative radiotherapy with or without adjuvant CT, and isocitrate dehydrogenase type 1 (*IDH1*) gene mutation status) were obtained from GSE16011 database as a training set (http://www.ncbi.nlm.nih.gov/geo/query/acc.cgi?acc=GSE16011), and the validation datasets include The Cancer Genome Atlas (TCGA) database (mRNA and RNAseq data) (http://cancergenome.nih.gov) and Repository for Molecular Brain Neoplasis Data (REMBRANDT) (http://caintegrator.nci.nih.gov/rembrandt). The RNAseq data were log2 transformed before analysis. A set of 78 B cell lineage-specific genes (Supplementary Table S1A) was adopted from a previous study [30].

### Statistical analysis and signature identification and validation

Patients alive for more than 90 days were eligible for the study because too short survival was more likely resulted from severe complication rather than glioma occurrence. In 232 glioma samples from GSE16011 dataset, there were 22 grade II tumors, 78 grade III tumors (including AA, AO, and AOA), and 132 GBMs. OS was calculated as the interval from the day of first surgery to death or the end of follow-up. Firstly, an unpaired two-tailed Student's *t* test was used to discriminate the expression level of each gene in the B cell lineage-specific gene set between each two of II-IV grade tumors. Secondly, the prognostic value of the differentially expressed gene (higher or lower than the median expression level) was calculated by the univariate Cox regression analysis with log-rank test by packages (survival) of R to get the corresponding Hazard Ratio (HR) and *p value* in four groups (grade II, III, IV, and HGG). Then the differentially expressed genes with significant prognostic value (*p <* 0.05) were selected after screened by the two steps. As a result, we identified five B cell-associated genes, which were then used to form a signature for prediction assessment and further validation in other two datasets (TCGA and REMBRANDT).

To test the power of the five gene signature for predicting clinical outcomes, a risk score formula for survival prediction was constructed according to a linear combination of the mRNA expression level of the five genes and weighted by the regression coefficient from the univariate Cox regression analyses (β) [65]. Based on the five gene signature, the risk score for each patient was calculated as follows:

Risk score = expr gene1 × βgene1 +expr gene2 × βgene2+… + expr gene5 × βgene5

According to this model, patients having high risk scores were expected to have poor OS. Patients of each grade in the training set were stratified into a high or low risk group by using the 50th percentile risk score as the cut off. Considering genes with multiple probes in the microarray settings, we chose the probe having a larger standard deviation (SD) and smaller β value, which is more likely to have a prognostic value with less likely to have a bias. This is a widely accepted method to filter genes with multiple probes by applying SD or median absolute deviation (MAD) [54]. We used the same β in the validation sets. The Kaplan–Meier method was used to discriminate overall survival by using the Mantel log-rank to assess the statistical significance between different groups with GraphPad Prism 6.0 statistical software.

To further annotate the biological insight of the high and low risk groups stratified by the signature, the differently expressed genes of HGGs were identified by significance analysis of microarray (SAM). Those genes with increased expression in tumors of high risk patients were used for Gene Ontology (GO) analysis in DAVID (http://david.abcc.ncifcrf.gov/). Gene Set Enrichment Analysis (GSEA) was downloaded from the Broad Institute (www.broadinstitute.org/gsea) for functional annotation. Heat maps of different grades of gliomas were constructed by Gene Cluster 3.0 and Gene Tree View software. The χ2 test was applied for statistical analysis of the correlation for two independent variables. Univariate and multivariate Cox analyses were performed using the Cox proportional hazard method; all the variables chosen were based on our clinical experience, which are related to prognosis. A two-sided *p value* of < 0.05 was regarded as significant.

## SUPPLEMENTARY MATERIALS FIGURES AND TABLE




