# Supplementary material for: Identification of a five B cell-associated gene prognostic and predictive signature for advanced glioma patients harboring immunosuppressive subtype preference
Source: Oncotarget. 2016 Oct 12;7(45):73971–83. doi: 10.18632/oncotarget.12605 (PMC5342028; doi:10.18632/oncotarget.12605)
Supplement: Supplementary file 1 [file oncotarget-07-73971-s001.pdf]

Identification of a five B cell-associated gene prognostic and predictive signature for advanced glioma patients harboring immunosuppressive subtype preference

Supplementary Materials

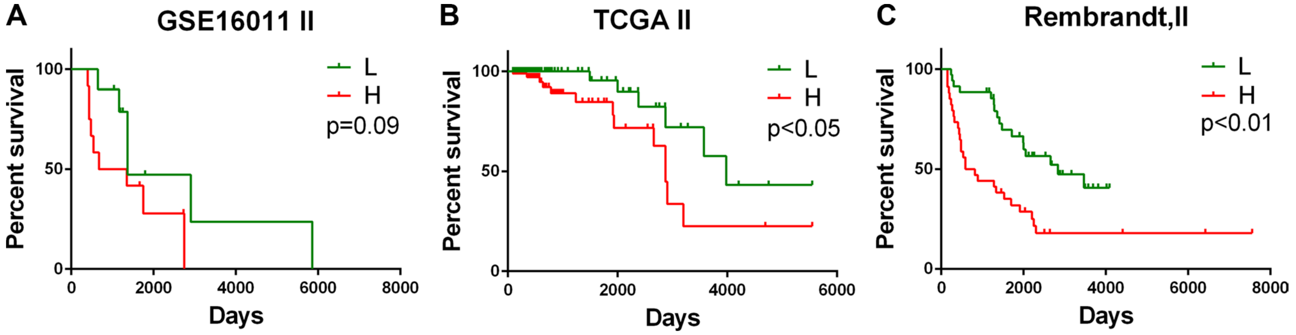

Supplementary Figure S1: Prognostic value of the five B cell-associated gene signature for grade II glioma patients in the three datasets. Patients in the low risk group showed a better prognosis than those in the high risk group according to the signature risk score in GSE16011 dataset (**A**), TCGA dataset (**B**), and Rembrandt dataset (**C**). L, low risk group; H, high risk group; II, WHO grade II.

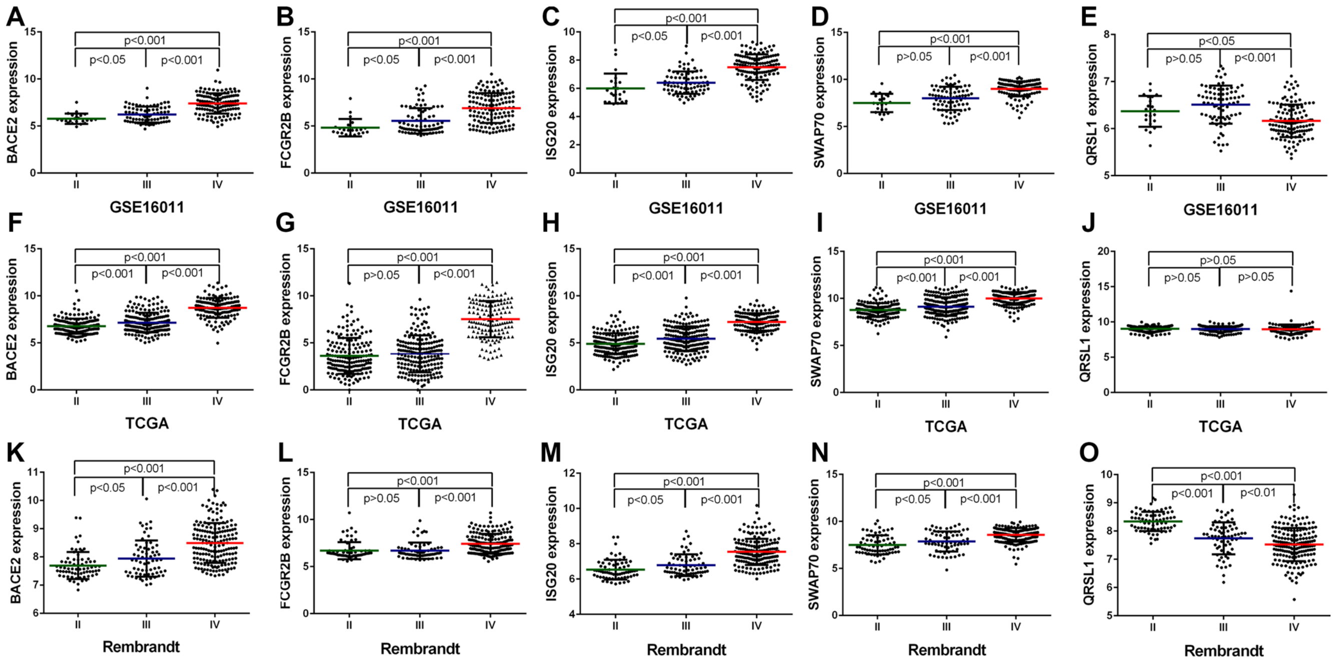

Supplementary Figure S2: Differential expression patterns of the five signature genes in glioma tumors of grade II, III, and IV in the three datasets. (**A–E**) GSE16011 dataset; (**F–J**) TCGA dataset; (**K–O**) Rembrandt dataset. Each spot represents the gene expression value of the individual tumor. Line in the middle was the mean expression value of each gene. II, WHO grade II; III, WHO grade III; IV, WHO grade IV.

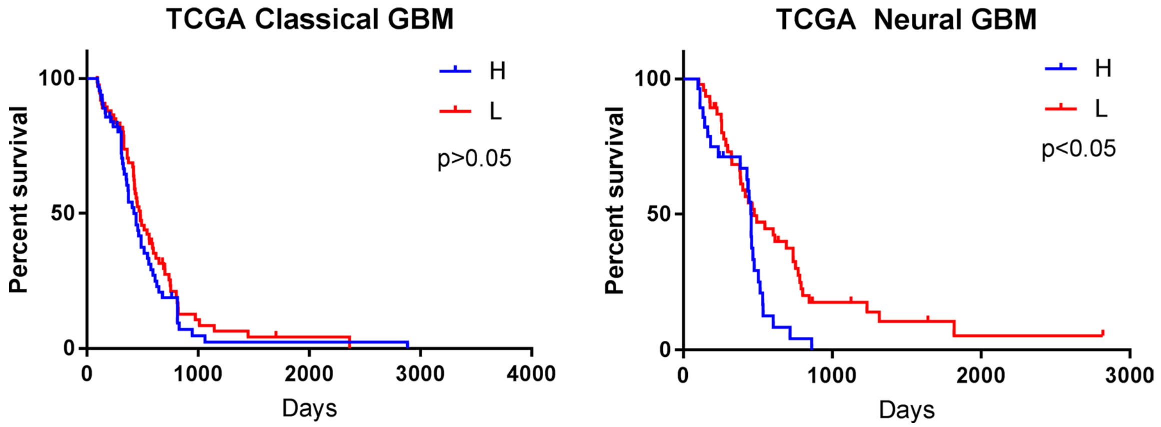

Supplementary Figure S3: Prognostic value of the five B cell-associated gene signature for GBM patients with Classical and Neural subtype tumors in TCGA dataset. Patients in the low risk group showed a better prognosis than those in the high risk group derived from Classical and Neural subtypes. L, the low risk group; H, the high risk group.

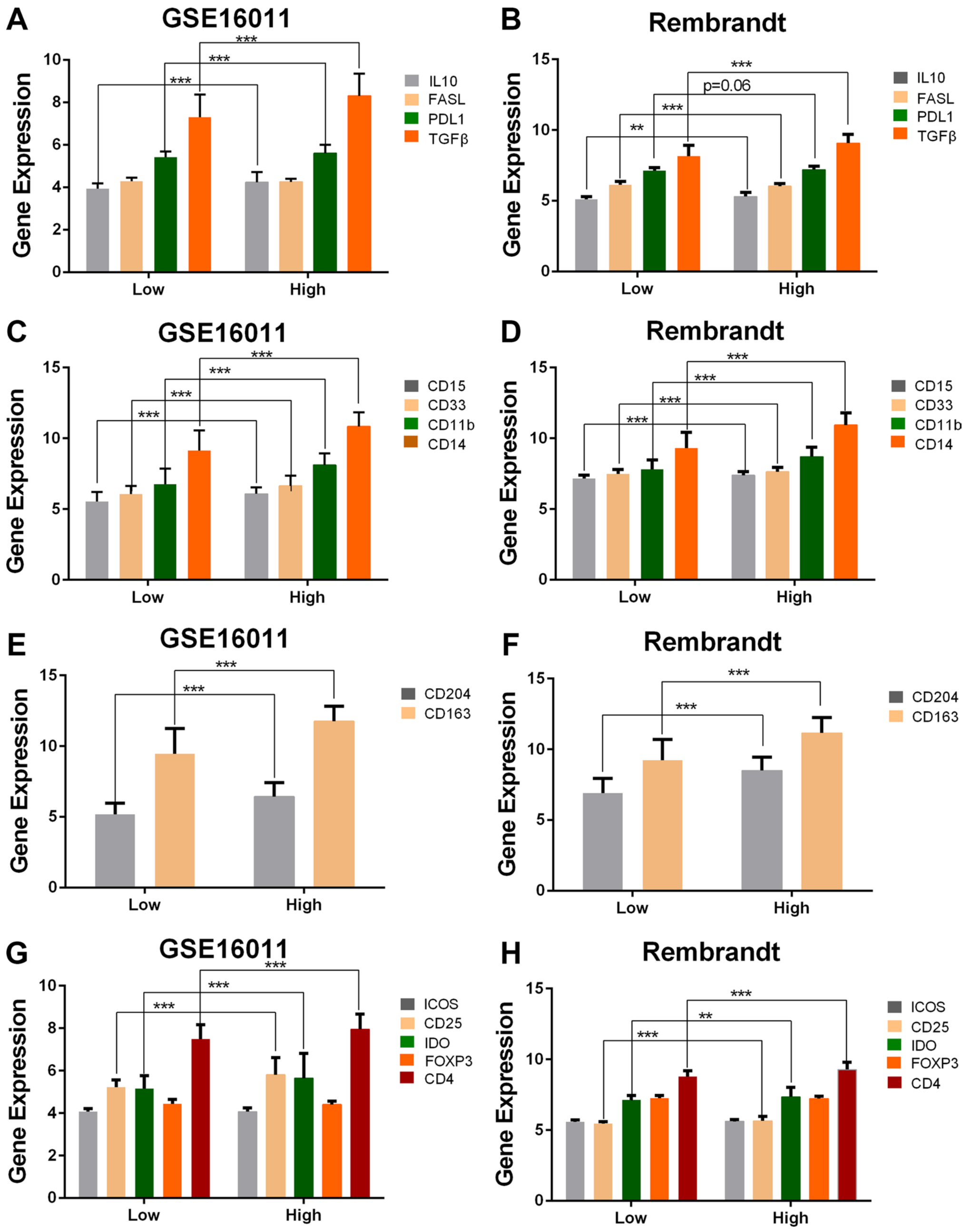

Supplementary Figure S4: Differential expression patterns of immunosuppressive factors and marker genes of immunosuppressive cells in GSE16011 (A, C, E and G) and Rembrandt (B, D, F and H) datasets. **P* < 0.05; ***P* < 0.01; ****P* < 0.001. Immunosuppressive factors: TGF-β, IL10, PDL1 (gene name *CD274*), and FASL (*FASLG*). Markers of immunosuppressive cells: MDSC markers (CD11b (*ITGAM*), CD14, CD15 (*FUT4*), CD33), M2 microglia/macrophage markers (CD68, CD163, CD204 (MSR1), CD206 (*MRC1*)), Treg cell markers (CD4, CD25 (*IL2RA*), ICOS, IDO1, FoxP3).

Supplementary Table S1: A gene set consists of 78 B cell-lineage specific genes. See Supplementary_ Table_S1
