# Supplementary material for: Identification of a five B cell-associated gene prognostic and predictive signature for advanced glioma patients harboring immunosuppressive subtype preference
Source: Oncotarget. 2016 Oct 12;7(45):73971–83. doi: 10.18632/oncotarget.12605 (PMC5342028; doi:10.18632/oncotarget.12605)
Supplement: Supplementary file 2 [file oncotarget-07-73971-s002.docx]

| **Supplementary Table S1A**  **A gene set consists of 78 B**  **cell-lineage specific genes.** | **Supplementary Table S1B-C**  **The overlapped differentially expressed genes between low and high risk groups in three microarray based datasets.** | | **Supplementary Table S1D GSE32423_IL7_VS_IL4_MEMORY_CD8_TCELL_DN**  **(Genes down-regulated in comparison of memory CD8 T**  **cells treated with IL7 versus those treated with IL4)** |
| --- | --- | --- | --- |
|  | **down-regulated**  **in high risk group** | **up-regulated**  **in high risk group** |  |
| ABCB4 | GPR123 | ABCC3 | ACOX3 |
| ACP5 | ZRANB1 | ACTN1 | ADH4 |
| ADAM19 | ZNF804A | ACTR3 | AGTRAP |
| ADAM28 | ZNF74 | ADAM12 | AIMP2 |
| ADK | ZNF711 | ADAP2 | ATG9A |
| BACE2 | ZNF681 | ADM | ATP5J2 |
| BACH2 | ZNF609 | ADPRH | ATP5O |
| BANK1 | ZNF540 | AEBP1 | AVEN |
| BIRC3 | ZNF25 | AGTRAP | BATF3 |
| BLK | ZNF238 | AIM1 | BOLA3 |
| CCR6 | ZMYND11 | AK2 | C12orf62 |
| CD180 | ZMIZ1 | ALOX5 | C14orf118 |
| CD19 | ZFYVE20 | ALOX5AP | C15orf17 |
| CD22 | ZCCHC14 | ANG | C17orf42 |
| CD24 | ZC3H12B | ANTXR2 | C19orf47 |
| CD37 | YPEL1 | ANXA1 | C1orf144 |
| CD40 | XKR4 | ANXA2 | C20orf3 |
| CD72 | WASF3 | ANXA4 | C21orf91 |
| CD79A | WASF1 | ANXA5 | C3orf38 |
| CD79B | WAC | APOBEC3C | C4B |
| CR2 | VIPR2 | APOBEC3G | C6orf64 |
| CXCR5 | UPF2 | ARHGAP18 | C9orf103 |
| EAF2 | UNC80 | ARHGAP29 | CA7 |
| EZR | UNC5A | ARPC1B | CCDC22 |
| FAIM3 | UNC13A | ARSD | CCDC77 |
| FAM30A | UBQLN4 | ARSJ | CCR10 |
| FAM46C | TUB | ASL | CDH5 |
| FCER2 | TTC3 | B3GNT5 | CEND1 |
| FCGR2B | TSPYL4 | BACE2 | CHCHD10 |
| FCRL2 | TOX3 | BCAT1 | CHURC1 |
| GGA2 | TOX | BCL2A1 | CIB1 |
| GPR18 | TNRC6C | BCL3 | CLU |
| HHEX | TNK2 | BIRC3 | CMKLR1 |
| HIST1H1C | TMOD2 | BST2 | CNDP2 |
| HLA-DOB | TMEM35 | C13orf18 | CNP |
| HLA-DPA1 | TMEM170B | C15orf48 | COL10A1 |
| HLA-DQA1 | TMEM151B | C17orf91 | COPA |
| HLA-DQB1 | TMCC1 | C1orf54 | COX8A |
| IFT57 | TM9SF3 | C1orf85 | DAXX |
| IGHA1 | THRA | C1QB | DBNL |
| IGHD | TET1 | C1R | DCTN5 |
| IGHG1 | TERF2 | C1RL | DDC |
| IGHM | TAF3 | C1S | DENND5A |
| IGKC | TACC2 | C21orf62 | DERA |
| IGL@ | SVOP | C21orf63 | DHODH |
| IGLJ3 | SUV420H1 | C2orf28 | DHX58 |
| IL4R | SUSD5 | C5AR1 | DLK1 |
| ISG20 | STXBP1 | C6orf115 | DMKN |
| KIAA0125 | STOX2 | C8orf4 | DMRTC2 |
| LOC652493 | ST8SIA3 | C9orf21 | DNLZ |
| LTB | SS18L1 | CALD1 | DUSP11 |
| MBD4 | SRGAP3 | CALU | EIF1AD |
| MS4A1 | SREBF2 | CAPG | EIF2S3 |
| NFATC1 | SPIN3 | CAPZA1 | ELF1 |
| NUP88 | SOX8 | CARD16 | ENSA |
| ODC1 | SOX6 | CASP1 | FAM156A |
| OSBPL10 | SOCS7 | CASP4 | FAM159B |
| P2RX5 | SOBP | CASP7 | FAM18A |
| PALM2-AKAP2 | SNX32 | CASP8 | FLYWCH2 |
| PDLIM1 | SNAP91 | CAST | FYB |
| PLEKHF2 | SMPD3 | CAV2 | G0S2 |
| PNOC | SMOC1 | CCDC109B | GABARAPL2 |
| POU2AF1 | SMARCC2 | CCL2 | GBP6 |
| QRSL1 | SLITRK5 | CD109 | GEMIN5 |
| RABEP2 | SLITRK1 | CD14 | GGT5 |
| RFX5 | SLIT1 | CD151 | GHR |
| RPS21 | SLC8A3 | CD163 | GLIPR2 |
| RRAS2 | SLC6A1 | CD164 | HLA-DQA1 |
| SP100 | SLC25A27 | CD300A | HSD3B7 |
| SP140 | SLC25A21 | CD44 | HSPD1 |
| SPIB | SLC22A17 | CD53 | IFIT1B |
| STAG3 | SLC1A6 | CD58 | IKZF3 |
| STAP1 | SLC16A9 | CD63 | ILF2 |
| SWAP70 | SIRT1 | CD97 | INPP4B |
| SYPL1 | SHD | CD99 | IRF4 |
| TCL1A | SHANK2 | CDCP1 | JAK3 |
| VPREB3 | SH3GL2 | CEBPB | KBTBD4 |
| ZNF395 | SERP2 | CEBPD | KCNS3 |
|  | SEMA4G | CFI | KLHL11 |
|  | SEC61A2 | CFLAR | KLK6 |
|  | SEC31B | CHI3L1 | KRTAP26-1 |
|  | SCRT1 | CHI3L2 | LARP1 |
|  | SCN3B | CHST2 | LCP1 |
|  | SCN3A | CLDN23 | LDLR |
|  | SCG3 | CLEC2B | LGALS3BP |
|  | SCAPER | CLEC5A | LRRC2 |
|  | SCAMP5 | CLEC7A | MAP3K8 |
|  | SCAI | CLIC1 | MAP6 |
|  | SBK1 | CMTM6 | MAP7D2 |
|  | SATB1 | COL1A2 | MCL1 |
|  | RUNDC3A | COL5A1 | MCTS1 |
|  | RUFY3 | COL5A2 | MDGA1 |
|  | RUFY2 | COL6A2 | METTL9 |
|  | RTN1 | COL6A3 | MGC23284 |
|  | RPS6KA5 | COL8A2 | MRPL12 |
|  | RPRD2 | COPZ2 | MRPL17 |
|  | RP1-177G6.2 | CPVL | MRPL36 |
|  | RNF165 | CRYZ | MRPS25 |
|  | RIPPLY2 | CSTA | MRPS26 |
|  | RIMS2 | CTBS | MRPS34 |
|  | RIMS1 | CTSA | MTIF3 |
|  | RICTOR | CTSB | MYBPC1 |
|  | RHBDL3 | CTSC | MYRIP |
|  | RGR | CTSL1 | NAA40 |
|  | RFTN2 | CTSS | NCAPG2 |
|  | REPS2 | CXCR4 | NCBP1 |
|  | RCOR2 | CYBA | NDST1 |
|  | RBM17 | CYTIP | NDUFB4 |
|  | RAPGEF4 | DCTD | NFIL3 |
|  | RAP2A | DDB2 | NFYC |
|  | RALGPS1 | DENND2D | NME1 |
|  | PTCHD2 | DIRAS3 | NTAN1 |
|  | PTCH1 | DOK1 | NTPCR |
|  | PPP3CB | DOK3 | NUDT1 |
|  | PLCXD2 | DPY19L1 | NUMB |
|  | PLCB4 | DPYD | NUP107 |
|  | PLCB1 | DYNLT3 | OR4C3 |
|  | PKNOX2 | EFEMP2 | PAM16 |
|  | PIP4K2B | ELF4 | PCDH17 |
|  | PID1 | EMILIN2 | PCGF2 |
|  | PHLPP1 | EMP1 | PCSK9 |
|  | PHF16 | EMP3 | PFKM |
|  | PHACTR3 | EMR2 | PGK2 |
|  | PDZD4 | ERI1 | PHF5A |
|  | PDE2A | F11R | PIGS |
|  | PCSK2 | F13A1 | PIH1D1 |
|  | PCGF2 | F3 | PINX1 |
|  | PATZ1 | FABP5 | PKN3 |
|  | PANK1 | FAH | PKNOX2 |
|  | PAK7 | FAM114A1 | PLBD1 |
|  | PAK3 | FAM129A | PLP2 |
|  | PABPC5 | FAM20C | PPM1L |
|  | OMG | FAM26F | PPP3CC |
|  | OLIG2 | FAM46A | PRKD3 |
|  | NTNG2 | FAS | PRPF31 |
|  | NSUN6 | FBP1 | PSMB3 |
|  | NRXN2 | FCER1G | PSMD14 |
|  | NRXN1 | FCGR2A | PSMD9 |
|  | NRSN1 | FCGR2B | PSME1 |
|  | NOLC1 | FCGR2C | PSPH |
|  | NOL4 | FKBP9 | PSRC1 |
|  | NMNAT2 | FN1 | RAB13 |
|  | NLGN2 | FNDC3B | RAB19 |
|  | NKAIN1 | FPR1 | RAP1GDS1 |
|  | NF1 | FSTL1 | RASGRP1 |
|  | NEU4 | FTL | RBKS |
|  | NET1 | FUCA1 | RBMS1 |
|  | NDRG3 | FUCA2 | RBP1 |
|  | NDRG2 | FXYD5 | RGNEF |
|  | NCAM1 | G0S2 | RGS1 |
|  | NBEA | GADD45A | RIPK1 |
|  | NAV1 | GALM | RIPK3 |
|  | NAP1L3 | GBP1 | RNPEP |
|  | NALCN | GCLM | RRP7A |
|  | MYT1 | GDF15 | SAR1B |
|  | MYST4 | GLB1 | SARS |
|  | MYCN | GLIPR1 | SDR42E1 |
|  | MXI1 | GLRX | SETD1A |
|  | MTSS1L | GLT25D1 | SHF |
|  | MTPAP | GMFG | SHOX2 |
|  | MTMR9 | GNG12 | SLC25A34 |
|  | MTA3 | GNG5 | SLC6A5 |
|  | MORN4 | GNS | SLC9A6 |
|  | MN1 | GPNMB | SLFN13 |
|  | MLLT6 | GPR65 | SLIRP |
|  | MLL | GPX8 | SMAD5 |
|  | MICAL3 | GSTK1 | SMARCE1 |
|  | MGEA5 | GUSB | SMC1A |
|  | MEX3B | H2AFJ | SNF8 |
|  | MEGF11 | HEBP1 | SNRPA |
|  | MDN1 | HEBP2 | SPATA5L1 |
|  | MDGA2 | HEXB | SPRR2A |
|  | MAST1 | HFE | SRGAP3 |
|  | MAPT | HMOX1 | SSRP1 |
|  | MAPK8IP3 | HRH1 | ST6GALNAC5 |
|  | MAPK8IP2 | HSD3B7 | STAMBP |
|  | MAP2 | HSPA5 | SUSD3 |
|  | LRRC4 | HSPA6 | SUV39H1 |
|  | LRRC20 | HSPB1 | TAMM41 |
|  | LRCH2 | IBSP | TGFBR3 |
|  | LOC283267 | ICAM1 | TGIF2 |
|  | LOC283174 | ICAM3 | TMEM104 |
|  | LOC254559 | IFI30 | TMEM140 |
|  | LOC157627 | IFITM2 | TMEM143 |
|  | LOC100128977 | IFITM3 | TMEM234 |
|  | LINGO1 | IFNGR2 | TMEM40 |
|  | LGR5 | IGFBP2 | TNFAIP3 |
|  | KPNA5 | IGFBP3 | TNNI3 |
|  | KLRC3 | IGFBP7 | TOR2A |
|  | KLF12 | IL15 | TRIM26 |
|  | KIF5C | IL8 | TRIM27 |
|  | KIF3C | IQGAP1 | TULP3 |
|  | KIF3A | IRAK2 | UFC1 |
|  | KIF21B | IRAK4 | UPB1 |
|  | KIAA1549 | IRF1 | UQCRQ |
|  | KIAA1543 | ISG20 | VCAM1 |
|  | KIAA1409 | ITGA5 | XPNPEP1 |
|  | KIAA1279 | ITGB1 | ZFHX3 |
|  | KIAA1211 | KCNE4 | ZHX3 |
|  | KIAA0427 | KDELC2 | ZMAT2 |
|  | KCNK3 | KDELR2 |  |
|  | KCNIP3 | KDELR3 |  |
|  | KCNIP2 | KIAA0040 |  |
|  | KCNB1 | KIAA0495 |  |
|  | KBTBD6 | KYNU |  |
|  | JPH4 | LAIR1 |  |
|  | JPH3 | LAMB1 |  |
|  | JMY | LAMC1 |  |
|  | JMJD1C | LAP3 |  |
|  | INA | LAPTM5 |  |
|  | IL1RAPL1 | LATS2 |  |
|  | IKZF5 | LCP2 |  |
|  | IDI1 | LDHA |  |
|  | HR | LEPRE1 |  |
|  | HNRNPH3 | LEPREL1 |  |
|  | HMX1 | LGALS1 |  |
|  | HMP19 | LGALS3 |  |
|  | HLF | LIF |  |
|  | HIPK2 | LILRB2 |  |
|  | HES6 | LITAF |  |
|  | HERC1 | LOC100132707 |  |
|  | HDAC5 | LOC154761 |  |
|  | HDAC4 | LOX |  |
|  | GRIK2 | LOXL1 |  |
|  | GRID1 | LOXL3 |  |
|  | GRIA4 | LTF |  |
|  | GRIA2 | LUM |  |
|  | GPSM1 | LY96 |  |
|  | GPRIN1 | LYN |  |
|  | GPR173 | LYZ |  |
|  | GNAO1 | MAN1C1 |  |
|  | GLCCI1 | MAP2K3 |  |
|  | GFRA1 | MBD2 |  |
|  | GDPD1 | METTL7B |  |
|  | GDAP1L1 | MGST2 |  |
|  | GDAP1 | MICA |  |
|  | GATS | MIR155HG |  |
|  | GABRB3 | MOXD1 |  |
|  | GABBR1 | MR1 |  |
|  | FXYD6 | MRC2 |  |
|  | FUT9 | MS4A4A |  |
|  | FSD1 | MS4A6A |  |
|  | FRY | MSN |  |
|  | FRAT1 | MSR1 |  |
|  | FLRT1 | MXRA8 |  |
|  | FLJ42627 | MYD88 |  |
|  | FGF13 | MYL12A |  |
|  | FGF12 | MYL6 |  |
|  | FERMT1 | MYOF |  |
|  | FCHSD2 | NAGA |  |
|  | FBXL16 | NAMPT |  |
|  | FBLL1 | NCF1C |  |
|  | FAM57B | NCF2 |  |
|  | FAM192A | NEK6 |  |
|  | FAM190B | NFKBIZ |  |
|  | FAM171A1 | NMI |  |
|  | FAM155A | NNMT |  |
|  | FAM13C | NPC2 |  |
|  | FAM133A | NRP1 |  |
|  | FAM123C | NTAN1 |  |
|  | FAM123A | OCIAD2 |  |
|  | FAM117B | OSMR |  |
|  | FAM110B | OSTC |  |
|  | EPHB1 | P4HA2 |  |
|  | ENHO | PARP9 |  |
|  | EML5 | PDIA4 |  |
|  | ELMO1 | PDIA5 |  |
|  | ELFN2 | PDLIM1 |  |
|  | ELAVL4 | PDLIM4 |  |
|  | ELAVL3 | PDPN |  |
|  | ELAVL2 | PGCP |  |
|  | EIF4EBP2 | PHLDA2 |  |
|  | DUSP26 | PIGT |  |
|  | DSCAML1 | PION |  |
|  | DSCAM | PLA2G5 |  |
|  | DRP2 | PLAU |  |
|  | DPYSL4 | PLBD1 |  |
|  | DPP10 | PLIN2 |  |
|  | DOK6 | PLK3 |  |
|  | DNM3 | PLOD1 |  |
|  | DLL3 | PLOD2 |  |
|  | DLL1 | PLP2 |  |
|  | DKFZp686O24166 | PLS3 |  |
|  | DIP2C | PLSCR1 |  |
|  | DHTKD1 | PMP22 |  |
|  | DENND5B | POLR2L |  |
|  | DDX25 | POSTN |  |
|  | DCX | PPCS |  |
|  | DACH2 | PPIC |  |
|  | CYP2E1 | PPP1R3B |  |
|  | CYFIP2 | PQLC3 |  |
|  | CXXC4 | PRDX4 |  |
|  | CXorf1 | PRSS23 |  |
|  | CUX2 | PTGER4 |  |
|  | CSTF2T | PTGS1 |  |
|  | CSNK1E | PTPN2 |  |
|  | CSMD3 | PTRF |  |
|  | CSMD1 | PTX3 |  |
|  | CRTAC1 | PVRL2 |  |
|  | CPLX1 | PYCARD |  |
|  | CPEB3 | PYGL |  |
|  | CNTN1 | RAB27A |  |
|  | CLIP3 | RAB32 |  |
|  | CLASP2 | RAB34 |  |
|  | CHGB | RAC2 |  |
|  | CHD7 | RAP2B |  |
|  | CHD3 | RBMS1 |  |
|  | CEP68 | RBPMS |  |
|  | CELSR3 | RCAN1 |  |
|  | CECR6 | RDH10 |  |
|  | CDK5R1 | REXO2 |  |
|  | CDH18 | RIPK1 |  |
|  | CCDC88A | RNASE2 |  |
|  | CCBL1 | RNASE4 |  |
|  | CBS | RRAS |  |
|  | CBLN1 | RUNX1 |  |
|  | CBARA1 | S100A10 |  |
|  | CAMSAP1L1 | S100A11 |  |
|  | CAMSAP1 | S100A13 |  |
|  | CADM2 | S100A16 |  |
|  | CACNG2 | S100A4 |  |
|  | CACNA1A | S100A6 |  |
|  | C9orf125 | S100A8 |  |
|  | C6orf168 | S100A9 |  |
|  | C6orf134 | SAT1 |  |
|  | C2orf27A | SDF4 |  |
|  | C17orf96 | SEC24D |  |
|  | C17orf69 | SECTM1 |  |
|  | C12orf53 | SERPINA1 |  |
|  | C12orf51 | SERPINA3 |  |
|  | C12orf34 | SERPINB1 |  |
|  | BZRAP1 | SERPINB6 |  |
|  | BTRC | SERPINE1 |  |
|  | BSN | SERPING1 |  |
|  | BRSK2 | SERPINH1 |  |
|  | BRD3 | SERTAD1 |  |
|  | BMS1P5 | SGMS2 |  |
|  | BCL7A | SHC1 |  |
|  | BCAN | SIGLEC7 |  |
|  | BAI3 | SLAMF8 |  |
|  | ATP9A | SLC10A3 |  |
|  | ATP8A1 | SLC11A1 |  |
|  | ATP6V1G2 | SLC12A7 |  |
|  | ATP2B2 | SLC16A3 |  |
|  | ATP1A3 | SLC22A18 |  |
|  | ATCAY | SLC25A24 |  |
|  | ASXL3 | SLC30A7 |  |
|  | ARL3 | SLC39A8 |  |
|  | ARID4A | SLC43A3 |  |
|  | ARHGEF9 | SLC7A7 |  |
|  | APBA2 | SOCS3 |  |
|  | ANKRD46 | SOD2 |  |
|  | ANKRD26 | SP100 |  |
|  | ANKRD13B | SP140L |  |
|  | ALDH5A1 | SPAG4 |  |
|  | ALCAM | SPATS2L |  |
|  | AKT3 | SPOCD1 |  |
|  | AKAP6 | SPP1 |  |
|  | AFF3 | SPPL2A |  |
|  | ADRBK2 | SQRDL |  |
|  | ADHFE1 | SRGN |  |
|  | ACVR2B | SSR3 |  |
|  | ACTL6B | ST8SIA4 |  |
|  | ACCN2 | STBD1 |  |
|  | ACADSB | STC1 |  |
|  | ACACA | STEAP3 |  |
|  | ABCC8 | SWAP70 |  |
|  | ABAT | TAGLN |  |
|  |  | TBC1D1 |  |
|  |  | TCIRG1 |  |
|  |  | TES |  |
|  |  | TGFB2 |  |
|  |  | TGFBI |  |
|  |  | THBD |  |
|  |  | THBS1 |  |
|  |  | TICAM2 |  |
|  |  | TIFA |  |
|  |  | TIMP1 |  |
|  |  | TLR2 |  |
|  |  | TMBIM1 |  |
|  |  | TMCO4 |  |
|  |  | TMED9 |  |
|  |  | TMEM176B |  |
|  |  | TMEM49 |  |
|  |  | TMEM71 |  |
|  |  | TMSB10 |  |
|  |  | TNFAIP6 |  |
|  |  | TNFAIP8 |  |
|  |  | TNFRSF11B |  |
|  |  | TNFRSF12A |  |
|  |  | TNFRSF1A |  |
|  |  | TOM1L1 |  |
|  |  | TPM4 |  |
|  |  | TRADD |  |
|  |  | TRAM1 |  |
|  |  | TREM1 |  |
|  |  | TRIM21 |  |
|  |  | TRIM38 |  |
|  |  | TRIM5 |  |
|  |  | TRIP6 |  |
|  |  | TRPV2 |  |
|  |  | TWSG1 |  |
|  |  | TYMP |  |
|  |  | TYROBP |  |
|  |  | UNC93B1 |  |
|  |  | UPP1 |  |
|  |  | VAMP5 |  |
|  |  | VAMP8 |  |
|  |  | VASN |  |
|  |  | VDR |  |
|  |  | VEGFA |  |
|  |  | VIM |  |
|  |  | WIPI1 |  |
|  |  | YIPF1 |  |
|  |  | ZNF217 |  |
|  |  | ZNF600 |  |
|  |  |  |  |
|  |  |  |  |
